# A redundant isoprenoid biosynthetic pathway supports *Staphylococcus aureus* metabolic versatility

**DOI:** 10.1128/mbio.00353-25

**Published:** 2025-06-30

**Authors:** Troy A. Burtchett, Elizabeth N. Ottosen, Tomotaka Jitsukawa, Momoko Kaneko, Miu Yasui, Jessica A. Lysne, Paige J. Kies, Jessica A. Bailey, Sean M. Thomas, Shingo Fujisaki, Neal D. Hammer

**Affiliations:** 1 Microbiology, Genetics, & Immunology, Biomedical & Physical Sciences Building, Michigan State University 3078, East Lansing, Michigan, USA; 2 Department of Biomolecular Science, Faculty of Science, Toho University, Funabashi, Chiba, Japan; The University of Arizona, Tucson, Arizona, USA

**Keywords:** isoprenoid, bacterial respiration, terminal oxidase, menaquinone, *Staphylococcus aureus*, farnesyl diphosphate, heptaprenyl diphosphate synthase, heme *o*, heme *a*, prenyl diphosphate synthase

## Abstract

Isoprenoids are ubiquitous molecules that serve as fundamental building blocks for life. In bacteria, isoprenoids are precursors for carotenoid pigments, respiratory cofactors, and essential sugar carrier lipids, such as lipid II. Isoprenoid synthesis initiates via condensation of the five-carbon (C_5_) precursors, isopentenyl diphosphate (IPP) and dimethylallyl diphosphate (DMAPP). This initial reaction condenses one DMAPP and two IPPs, resulting in C_15_ farnesyl diphosphate (FPP), an intermediate that is sequentially elongated with IPP. FPP is thought to be synthesized exclusively by the prenyl diphosphate synthase (PDS), IspA. In *Staphylococcus aureus*, *ispA* mutants lack the golden carotenoid pigment, staphyloxanthin. The fact that *ispA* can be inactivated in *S. aureus* and other bacteria is surprising given the reliance of lipid II on FPP and supports the hypothesis that an additional enzyme produces the critical isoprenoid precursor. We isolated pigmented *ispA* suppressor mutants harboring single-nucleotide polymorphisms within a second PDS-encoding gene, *hepT*, suggesting that HepT and IspA have overlapping roles in *S. aureus* isoprenoid synthesis. Subsequent work determined that IspA and HepT support metabolic versatility, as a *hepT ispA* double mutant fails to aerobically respire partially due to a lack of prenylated heme cofactors. The finding that a *hepT ispA* double mutant is viable supports a model whereby a third PDS compensates in the absence of *ispA* and *hepT* to produce lipid II precursors. Lastly, we show that *ispA* and *hepT* mutants exhibit colonization defects in a murine model of systemic infection, demonstrating that isoprenoid biosynthesis is a potential drug target for combating *S. aureus*.

**IMPORTANCE:**

Isoprenoid synthesis is an essential process that is presumed to be initiated by the prenyl diphosphate synthase (PDS), IspA. However, our understanding of this pathway is incomplete considering that *ispA* mutants have been described in several bacterial species, leaving the mechanism for isoprenoid synthesis initiation uncertain in these genetic backgrounds. Using the opportunistic pathogen *Staphylococcus aureus*, we demonstrate that a second PDS, HepT, supports the production of isoprenoid-dependent molecules in the absence of IspA. Importantly, we show that mutants deficient for either IspA or HepT display colonization defects in a murine model of systemic infection. Furthermore, the simultaneous mutation of *hepT* and *ispA* is tolerated in *S. aureus* and suggests the presence of a third PDS capable of initiating isoprenoid synthesis. This study establishes PDSs as viable targets for the treatment of *S. aureus* infections and provides novel insights into the redundant nature of isoprenoid synthesis in this pathogen.

## INTRODUCTION

Isoprenoid synthesis is a highly conserved process present in virtually all living organisms and begins with the production of the universal five-carbon (C_5_) precursors, isopentenyl diphosphate (IPP) and dimethylallyl diphosphate (DMAPP) (1, 2). Depending on the organism, IPP and DMAPP can be generated by either the methyl erythritol phosphate pathway, which is active in most bacteria, or the mevalonate pathway utilized by animals, archaea, and some gram-positive bacteria (3, 4). An initial condensation reaction of one DMAPP and one IPP produces geranyl diphosphate (GPP), to which a second IPP is immediately added to generate farnesyl diphosphate (FPP), a C_15_ isoprenoid that serves as a substrate for downstream cellular pathways (2). This initial condensation reaction and subsequent elongation reactions that add IPP units in a stepwise fashion are carried out by enzymes, called prenyl diphosphate synthases (PDSs) (2). Generally, bacteria harbor three PDSs: a short-chain PDS that catalyzes the initial condensation reaction between IPP and DMAPP to generate C_10_ GPP and then FPP; a medium-chain PDS that uses FPP and several additional IPP to produce medium-chain length isoprenoids; and a long-chain PDS that also uses FPP and IPP to generate long-chain isoprenoids (5–11). Medium-chain isoprenoids range in length from C_35_ to C_50_ and support the production of quinone respiratory cofactors (12, 13). Long-chain isoprenoids are C_55_ or greater and in bacteria include undecaprenyl phosphate (Und-P), which serves as a scaffold onto which glycan units are added to generate lipid II, an indispensable metabolite required for generating peptidoglycan (13, 14). Lipid II is essential for cell viability and the target of several classes of antibiotics (14–17). Therefore, a greater understanding of the isoprenoid synthesis in bacteria will reveal fundamental mechanisms by which critical isoprenoid precursors are allocated to support cell envelope maintenance and metabolism.

In the gram-positive pathogen *Staphylococcus aureus*, FPP is a substrate for three reactions: condensation, elongation, or prenylation, the covalent addition of an isoprenoid directly to a substrate (Fig. S1). The condensation of two FPPs generated by the short-chain PDS IspA is catalyzed by CrtM to generate dehydrosqualene, a C_30_ precursor for staphyloxanthin, the membrane-localized carotenoid antioxidant that gives the pathogen its distinctive golden color (18). As such, *ispA* and *crtM* mutants are not pigmented (19, 20). Elongation reactions add IPP to FPP in a stepwise fashion, and at least two elongation reactions are active in *S. aureus*. The first is catalyzed by UppS, the enzyme that produces long-chain undecaprenyl diphosphate (Und-PP). Und-PP is the precursor of the sugar carrier lipid, Und-P, that transports polysaccharides across the cytoplasmic membrane. Extracytoplasmic saccharide-based polymers generated by *S. aureus* include peptidoglycan, wall teichoic acid (WTA), and in some strains, capsular polysaccharide (CP) (14, 21, 22). Importantly, each of these polysaccharide cell surface structures is either essential (peptidoglycan) or contributes to virulence (WTA and CP), making UppS an essential enzyme and a high-priority target for therapeutic intervention (14, 23–26). Given the essentiality of UppS, it is surprising that the enzyme which produces the FPP substrate for its activity, IspA, is dispensable. Mutants lacking functional *ispA* have been generated in several different bacterial species, including *S. aureus*, *Escherichia coli*, *Pseudomonas aeruginosa*, and *Bacillus subtilis* (27–31). In *S. aureus*, *ispA* mutants produce reduced quantities of FPP compared to wild type (WT), indicating that at least one other PDS can produce FPP (27). Clarifying the PDS enzymes that generate UppS substrates will provide new knowledge for how the critical peptidoglycan lipid carrier is synthesized.

The second elongation is predicted to be carried out by HepT. *B. subtilis* HepT and the *E. coli* HepT homolog, IspB, produce the medium-chain isoprenoids used in menaquinone (MK) and ubiquinone (UQ) syntheses (8, 29, 32–35). MK and UQ are essential electron carriers in the electron transport chain. *S. aureus* produces MK, of which three species are synthesized that differ in the length of the isoprenoid group: MK-7, MK-8, and MK-9 (36, 37). The number refers to the C_5_ isoprenoid moieties; thus, MK-7, MK-8, and MK-9 have chain lengths of C_35_, C_40_, and C_45_, respectively. Of note, some strains of *S. aureus* harbor HepT alleles that result in the production of a C_50_ MK-10 (36), indicating that different *hepT* alleles influence the length of its isoprenoid product. These findings support a model whereby *S. aureus* HepT is required to generate MK; however, direct experimental evidence for this function is lacking.

MK is a required cofactor for aerobic respiration, and *S. aureus* mutant strains impaired for MK synthesis are restricted to fermentative metabolism. This leads to a distinctive phenotype, called the small colony variant (SCV), which coincides with increased antibiotic resistance (37–40). In fact, MK auxotrophs are among the most common types of SCVs isolated from cystic fibrosis patients (41). Aminoglycoside treatment selects for SCVs because the antibiotic requires the proton motive force (PMF) to enter the cell (42–44). In response, *S. aureus* develops inactivating mutations in the MK synthesis pathway, switching to fermentation and reducing the PMF (45). Notably, an *S. aureus hepT* mutant exhibited increased pigmentation, but growth phenotypes were not reported (20); therefore, it is unclear whether the mutation of *hepT* induces the SCV phenotype. Additionally, whether HepT participates in other isoprenoid-dependent pathways is uncertain, as HepT in *B. subtilis* and IspB in *E. coli*, *Acinetobacter baumannii*, and *Corynebacterium glutamicum* are essential, implying medium-chain PDSs may function beyond MK and UQ syntheses (35, 46–48). In fact, loss of heptaprenyl diphosphate synthase activity in *B. subtilis* was only tolerated when the mutant was cultured in a medium that supports cell wall-free L-form growth, revealing a potential role for HepT in cell wall synthesis (49). The fact that *HepT* mutants are viable in *S. aureus* suggests that unique or redundant isoprenoid biosynthetic pathways exist in this species and presents an opportunity to study the physiological consequences of HepT inactivation, which has not been possible in other bacterial species.

Prenylation, the third FPP-dependent reaction, also supports aerobic respiration through the covalent attachment of FPP to heme *b* to produce the modified heme cofactors, hemes *o* and *a*. Both prenylated heme cofactors are capable of supporting the activity of the major terminal oxidase, QoxABCD (50–52). Therefore, prenylation is essential for one of the two enzymes that perform the last step of aerobic respiration, the reduction of O_2_ to H_2_O (51). A second terminal oxidase, CydAB, functions in addition to QoxABCD, leading to a branched respiratory chain. The CydAB activity is not dependent on heme *o* or *a* (51, 53). Whether IspA produces FPP needed to generate heme *o* or *a* is unclear. The *ispA* mutant exhibits slightly increased resistance to aminoglycosides gentamicin and kanamycin, as well as a reduced ATP production, suggesting that the electron transport chain activity is attenuated (27). However, whether IspA provides the isoprenoid precursors required to generate the modified heme cofactors needed for the QoxABCD activity has not been directly demonstrated. Overall, resolving the contributions of IspA and HepT toward generating isoprenoid metabolites in *S. aureus* will provide novel insight into three major pathways: carotenoid pigment production, cell envelope maintenance, and respiration.

In this study, we show that isoprenoid synthesis supports metabolic versatility in *S. aureus* by demonstrating that IspA and HepT preferentially contribute to carotenoid pigment and MK synthesis, respectively. Either enzyme is sufficient for the generation of hemes *o* and *a*, indicating that both can provide the FPP precursor needed for heme prenylation. The basis for this conclusion is the finding that a *hepT ispA* mutant is viable but restricted to fermentative metabolism despite supplementation with the C_20_ MK analog, MK-4. These results support a model whereby a third enzyme generates FPP that is specifically used to produce Und-PP for peptidoglycan synthesis. Additional experimentation focused on chemically complementing MK deficiency using MK-4 and demonstrated that the shorter MK analog promotes QoxABCD but not CydAB activity. This result suggests that the MK isoprenoid chain length plays an important role in the CydAB function, indicating that different MK species modulate the activity of the branched respiratory chain. Lastly, we show that *ispA* or *hepT* mutants exhibit colonization defects in a murine model of systemic infection, providing evidence that the isoprenoid biosynthetic pathway is a potential drug target. Overall, these results support a revised model of *S. aureus* isoprenoid synthesis whereby precursors generated by specific enzymes are used to produce staphyloxanthin, MK, or Und-P, but prenylation of heme can be achieved using precursor from either IspA or HepT. These data show that redundancy within the isoprenoid synthesis pathway of *S. aureus* supports the metabolic versatility and virulence of this important human pathogen.

## RESULTS

### Pigmented *ispA* suppressor mutants contain nonsynonymous gain-of-function mutations in *hepT*

**Fig 1:**
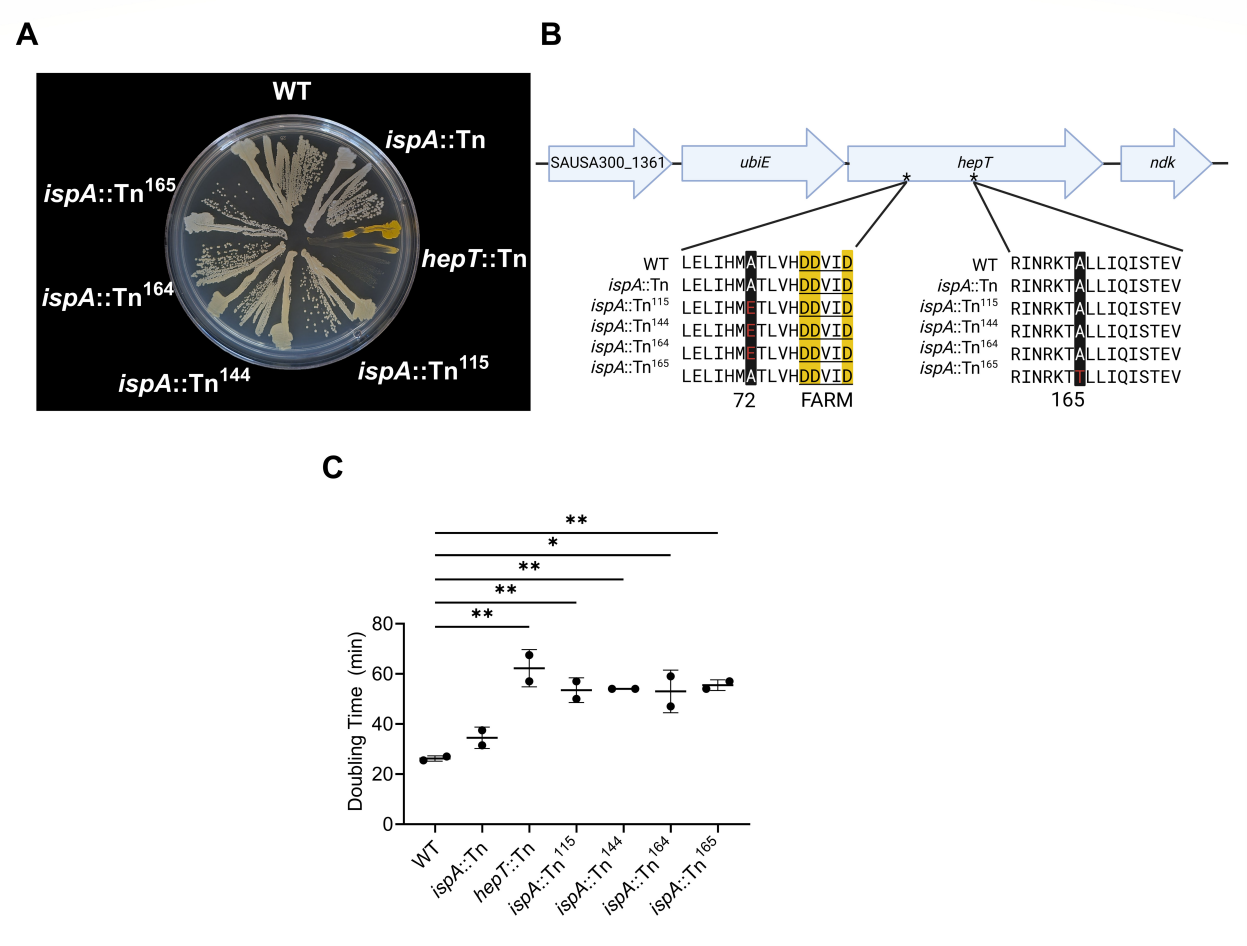
Pigmented *ispA*::Tn suppressors harbor SNPs within *hepT*. (**A**) Indicated strains were streaked on TSA and incubated overnight. (**B**) *hepT* is predicted to be operonic with SAUSA300_1361 and *ubiE* but not *ndk*. Multiple alignment of the HepT amino acid sequence encoded by the indicated strains. Conserved aspartic acid residues that comprise the first aspartic acid-rich motif (FARM) domain are highlighted in yellow. Black-highlighted residues represent the amino acid position that was altered due to acquired mutations in the *ispA*::Tn gentamicin-resistant mutants. (**C**) Doubling times of the indicated strains determined after incubation in TSB. Error bars represent one standard deviation from the mean. Statistical significance was determined via one-way ANOVA. * and ** represent *P*-values of <0.05 and <0.01, respectively.

A previous study reported that *ispA* mutants lack pigmentation and exhibit increased resistance to gentamicin (27). We sought to recapitulate these results using an agar-based assay whereby 10^8^ CFU/mL *ispA*::Tn mutant cells were spread onto a tryptic soy agar (TSA) plate supplemented with 3 µg/mL gentamicin, yielding at least 100 isolated resistant colonies per plate. Across three independent trials, the total quantity of gentamicin-resistant colonies ranged between 10^3^ and 10^4^ CFU/mL. Unexpectedly, pigmented resistant colonies could be observed at a rate of 0.04% among the nonpigmented resistant colonies across three independent biological replicates. Pigmented colonies demonstrated WT-like levels of staphyloxanthin production, indicating the presence of suppressor mutations (Fig. 1A). Whole-genome sequencing analysis revealed nonsynonymous mutations in the gene encoding heptaprenyl diphosphate synthase, *hepT* (Table S1). Notably, three resistant pigmentation suppressor mutants (*ispA*::Tn^115^, *ispA*::Tn^144^, and *ispA*::Tn^164^) isolated from three different trials shared the same A72E amino acid change five residues preceding the **f**irst **a**spartate-**r**ich **m**otif (FARM) (Fig. 1B). This amino acid position is highly conserved in IspA and HepT homologs (54) and has been implicated in determining the chain length of the isoprenoid product (55). The nonpigmented gentamicin-resistant control isolate harbored an amino acid change 93 amino acids downstream of the HepT FARM domain (A165T). Given the putative activity of *S. aureus* HepT, it is likely that a *hepT* mutant does not produce MK and therefore cannot perform respiration, resulting in the observed gentamicin resistance. Consistent with this, *hepT*::Tn has a slower growth rate compared to WT (Fig. 1C), as it likely relies on fermentation to generate energy. Growth rate analysis of *ispA*::Tn gentamicin-resistant colonies showed similar doubling times to *hepT*::Tn (Fig. 1C), suggesting that the *hepT* mutations likely lead to reduced production of the medium-chain isoprenoids necessary for MK synthesis. In fact, the altered FARM domain of HepT^A72E^ increases in similarity to the FARM domain of other PDSs that are known to synthesize short-chain isoprenoids (55) and might be performing an IspA-like role in producing FPP needed for pigmentation.

### Disruption of isoprenoid synthesis leads to downstream perturbations in the abundance of isoprenoid-derived metabolites

**Fig 2:**
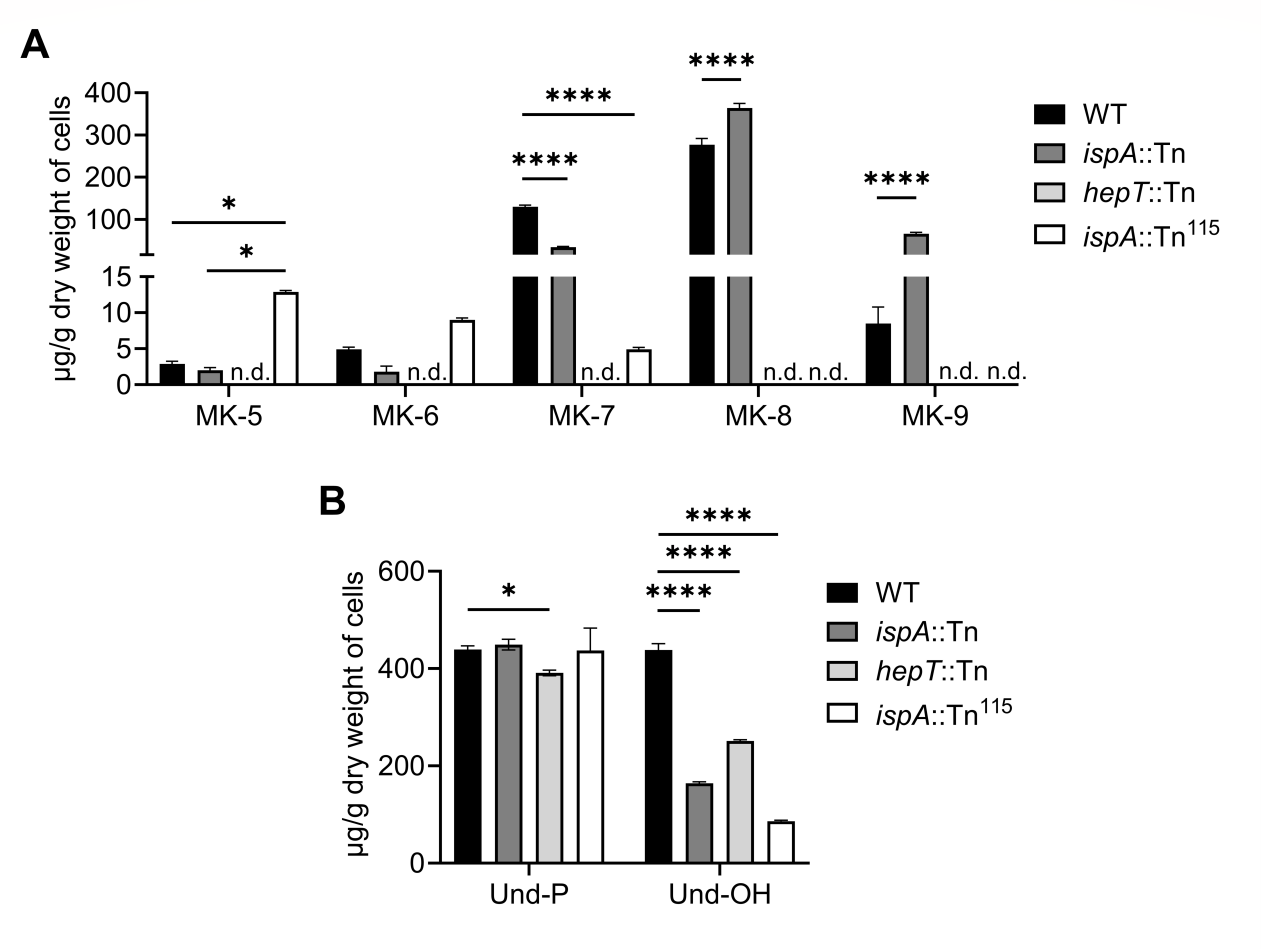
Mutating the PDS enzymes IspA and HepT alters the abundance of medium- and long-chain isoprenoid-containing metabolites. (**A**) High-performance liquid chromatography (HPLC) analysis of MKs extracted from whole cells. Error bars represent one standard deviation from the mean. Statistical significance was determined by two-way ANOVA with Tukey correction. * and **** represent *P*-values of <0.05 and <0.0001, respectively. n.d. (not detected) represents samples for which no MK was detected. (**B**) HPLC analysis of undecaprenyl phosphate (Und-P) and undecaprenol (Und-OH) extracted from whole cells. Statistical analysis was determined by two-way ANOVA with Tukey correction. * and **** represent *P*-values of <0.05 and <0.0001 respectively. Data presented in panels A and B are the average of three independent biological replicates performed in triplicate.

To determine the roles of IspA and HepT in the generation of downstream isoprenoid-containing metabolites, MK species undecaprenyl phosphate (Und-P) and undecaprenol (Und-OH) were quantified via high-performance liquid chromatography (HPLC). The primary MK species produced by WT was MK-8, followed by MK-7 and MK-9 (Fig. 2A), which is consistent with previous studies (36, 37). However, low levels of MK-5 and MK-6 were also detected in WT, which, to the best of our knowledge, is the first report of *S. aureus* producing these MK species. The mutation of *ispA* resulted in increased production of longer-chain MKs with a significantly higher production of MK-8 and MK-9 but a decreased production of MK-7, while MK-5 and MK-6 were not altered compared to WT. Notably, *hepT*::Tn did not produce detectable levels of any MK species. Consistent with the hypothesis that the HepT^A72E^ variant generates shorter-chain isoprenoids, *ispA*::Tn^115^ produced significantly higher levels of MK-5 compared to WT, whereas MK-8 and MK-9 were not detected, and MK-7 was also significantly reduced (Fig. 2A). Additionally, *hepT*::Tn contained significantly lower amounts of the lipid II precursor Und-P compared to WT (Fig. 2B). All of the mutants also demonstrated significantly lower quantities of Und-OH compared to WT, indicating both IspA and HepT may contribute to the production of precursors necessary for long-chain isoprenoid biosynthesis.

### Simultaneous inactivation of *hepT* and *ispA* induces a small colony variant phenotype that is unresponsive to MK-4 supplementation

**Fig 3:**
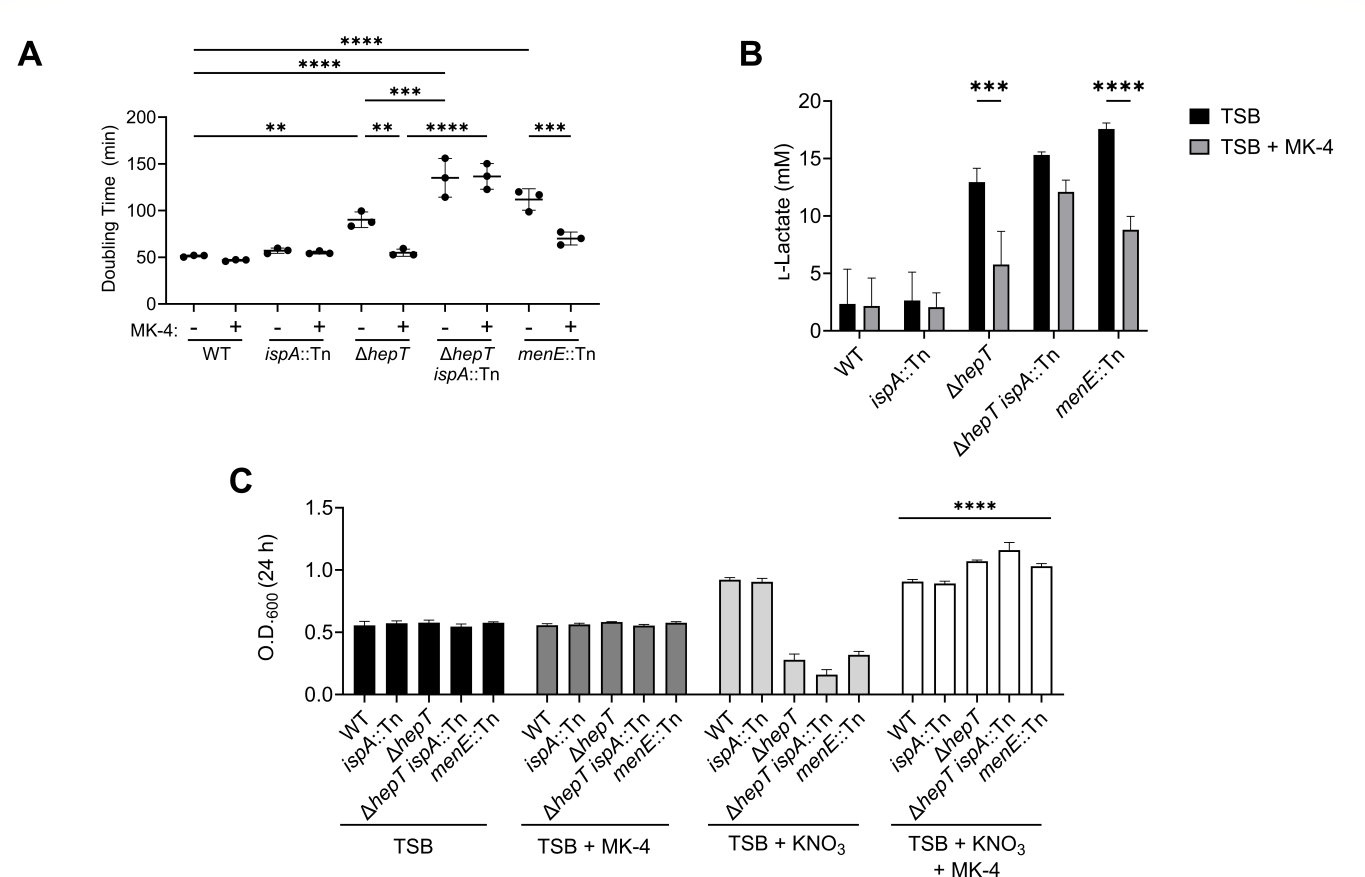
Simultaneous inactivation of *hepT* and *ispA* impairs aerobic respiration. (**A**) Doubling times of the indicated strains in TSB supplemented with or without 12.5 µM MK-4. Error bars represent one standard deviation from the mean. Statistical significance was determined via one-way ANOVA. **, ***, and **** represent *P*-values of <0.01, <0.001, and <0.0001, respectively. (**B**) The concentration of excreted l-lactate was determined from cell-free supernatants of the indicated strain. Statistical significance was determined by two-way ANOVA with Bonferroni correction. Error bars represent one standard deviation from the mean. *** and **** represent *P*-values of <0.001 and <0.0001, respectively. (**C**) Anaerobic stationary-phase optical density at 600 nm after 24 h incubation of the indicated strains cultured in TSB, TSB supplemented with MK-4, TSB supplemented with KNO_3_, or TSB supplemented with both KNO_3_, and MK-4. Error bars represent one standard deviation from the mean. Statistical significance was determined via two-way ANOVA comparing strains supplemented with MK-4 and KNO_3_ to strains lacking supplementation (TSB) or supplemented with MK-4. **** represents a *P*-value of <0.0001.

A consequence of inactivating *ispA* in *S. aureus* is reduced quantities of FPP, supporting a model whereby a second enzyme generates this indispensable precursor (27). Given that IspA and HepT are the only two enzymes known to contain polyprenylsynthetase FARM domains in *S. aureus*, we hypothesized that the simultaneous inactivation of both enzymes would result in synthetic lethality. Given that *hepT* is predicted to be the terminal gene in a three-gene operon and is in close proximity to *ndk* (Fig. 1B), an in-frame deletion was generated to reduce the possibility of polar effects. The Δ*hepT* mutant was subsequently transduced with φ85 propagated on the *ispA*::Tn mutant to yield the Δ*hepT ispA*::Tn double mutant. Surprisingly, Δ*hepT ispA*::Tn was viable but displayed a significantly increased doubling time compared to the WT and Δ*hepT* (Fig. 3A). The supplementation of the growth medium with MK-4 decreased the doubling time of Δ*hepT* and a *menE*::Tn control SCV to WT-like levels, indicating that respiration was restored in these mutants. This result shows that the slower growth rate observed in these mutants is due to a lack of MK that restricts metabolism to fermentation. However, the MK-4 supplementation failed to reduce the doubling time of Δ*hepT ispA*::Tn, indicating that this strain is unable to aerobically respire. Genetic complementation of Δ*hepT ispA*::Tn with plasmid-encoded *hepT* or *ispA* restored pigmentation, doubling time, and stationary-phase optical density at 600 nm (O.D._600_) to that of the respective single mutant (Fig. S2). These results confirm that the SCV phenotype observed in Δ*hepT ispA*::Tn is not due to second site mutations. Having the Δ*hepT ispA*::Tn mutant in hand allowed us to investigate the effects of the *hepT*^A72E^ allele independent of either *ispA* or *hepT*. Consistent with the isolation of the *hepT*^A72E^ allele in the pigmentation suppressor mutants, the allele partially restored pigmentation of Δ*hepT ispA*::Tn (Fig. S3A). However, the doubling time and the stationary-phase O.D._600_ remained similar to the empty vector control (Fig. S3B and C). These results demonstrate that expressing HepT^A72E^ does not complement the growth of the double mutant and provide an explanation for why this allele increases gentamicin resistance.

Given the hyperpigmentation phenotype of Δ*hepT* and the importance of *ispA* in staphyloxanthin production, we tested whether loss of the antioxidant carotenoid pigment was responsible for the respiration defect. A Δ*hepT crtM*::Tn double mutant was constructed and exhibited a WT-like colony size compared to the SCV variant phenotype of Δ*hepT ispA*::Tn (Fig. S4A). Additionally, MK-4 supplementation restored the doubling time and the stationary-phase O.D._600_ of Δ*hepT crtM*::Tn (Fig. S4B and C), demonstrating that loss of pigmentation is not responsible for the Δ*hepT ispA*::Tn SCV phenotype.

*S. aureus* predominantly produces lactate as a major fermentation product (53, 56). To confirm that Δ*hepT ispA*::Tn is restricted to fermentation, we quantified the lactate production of strains grown in tryptic soy broth (TSB) or TSB supplemented with MK-4. WT and *ispA*::Tn produced low amounts of l-lactate, regardless of MK-4 supplementation (Fig. 3B), indicating that respiration is the primary energy-generating pathway in these strains. The *menE*::Tn control SCV produced high levels of l-lactate, which were significantly reduced upon MK-4 supplementation. This is similar to Δ*hepT* and demonstrates that MK-4 induces respiration in these mutants. However, Δ*hepT ispA*::Tn produced high levels of l-lactate with or without MK-4 supplementation (Fig. 3B). Together, these results demonstrate that Δ*hepT ispA*::Tn is not capable of aerobic respiration.

Next, we sought to determine whether the failure of Δ*hepT ispA*::Tn to respond to MK supplementation is due to a general respiration deficiency or if it is specific to aerobic respiration. To test this, Δ*hepT ispA*::Tn was cultured anaerobically in the presence or absence of MK-4 and the alternative terminal electron acceptor, nitrate (KNO_3_), as *S. aureus* is capable of utilizing nitrate for anaerobic respiration (57, 58). WT, *ispA*::Tn, Δ*hepT*, Δ*hepT ispA*::Tn, and *menE*::Tn proliferated to similar end-point optical densities (Fig. 3C). The addition of the terminal electron acceptor, KNO_3_, increased the stationary-phase, end-point optical density of WT and the *ispA*:Tn mutant, which produce MK and are, therefore, capable of anaerobically respiring. Interestingly, the addition of KNO_3_ had an inhibitory effect on Δ*hepT*, Δ*hepT ispA*::Tn, and *menE*::Tn. Nitrate is often used as a preservative in food to prevent microbial growth (59), and we suspect nitrate may be inhibiting the proliferation of strains that cannot utilize this compound to anaerobically respire. Supplementation with both MK-4 and KNO_3_ increased the stationary-phase, end-point optical density of all strains, including Δ*hepT ispA*::Tn (Fig. 3C). Together, these data show that Δ*hepT ispA*::Tn is capable of anaerobic respiration via supplementation with MK-4, revealing that the SCV phenotype is specific to aerobic respiration.

### QoxABCD activity is impeded in the Δ*hepT ispA*::Tn double mutant due to loss of prenylated heme cofactors

**Fig 4:**
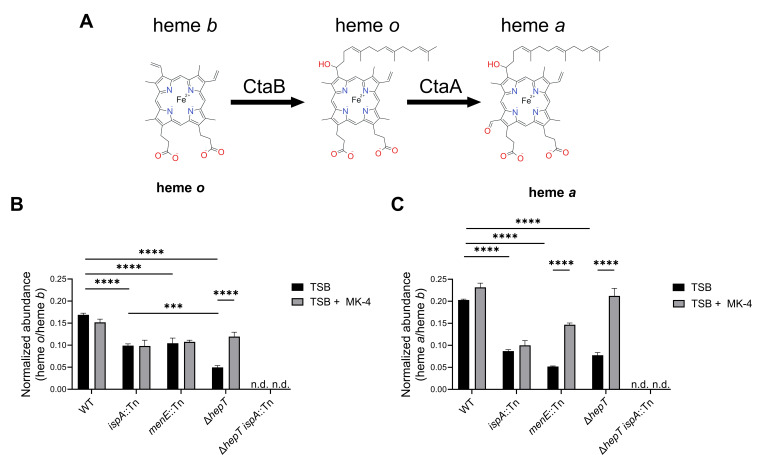
HepT and IspA contribute to the production of prenylated heme cofactors. (**A**) An illustration of the prenylated heme synthesis pathway beginning with heme *b*. Heme *b* farnesylated by CtaB generating heme *o*. Heme *o* is modified by the addition of a carbonyl group to produce heme *a*. (**B and C**) High-performance liquid chromatography analysis of hemes extracted from whole cells. Heme *b* was used as an internal standard to which hemes *o* (**B**) and *a* (**C**) were normalized for each sample. Data are the average of three independent biological replicates performed in triplicate. Statistical significance was determined via two-way ANOVA. *** and **** represent *P*-values of 0.001 and 0.0001, respectively.

*S. aureus* utilizes a branched aerobic respiratory chain consisting of the QoxABCD and CydAB terminal oxidases, both of which require heme for their activity (51, 53). The finding that Δ*hepT ispA*::Tn respires anaerobically but not aerobically supports the hypothesis that both QoxABCD and CydAB are impeded in this strain. The QoxABCD activity is dependent on prenylated heme cofactors, which are generated via the addition of FPP onto heme *b* by CtaB to produce heme *o*. Heme *o* is further modified by the addition of a carbonyl group by CtaA to produce heme *a* (Fig. 4A). While QoxABCD requires prenylated hemes (heme *o* or *a*) for its activity, CydAB functions with heme *b* as the sole heme cofactor (51). To determine whether prenylated heme cofactors are produced in Δ*hepT ispA*::Tn, hemes were extracted from overnight cultures and quantified via HPLC. In each strain, heme *b* was detected and used as an internal standard to normalize heme *o* and *a* abundance across samples. The mutation of *ispA* leads to a significant decrease in heme *o* and *a* abundance compared to WT (Fig. 4B and C). Interestingly, the disruption of MK synthesis via the mutation of *menE* also leads to a significant decrease in hemes *o* and *a* compared to WT and suggests that modified heme cofactor production is reduced during fermentative growth. Supplementation of the *menE*::Tn mutant with MK-4 partially restores heme *a* abundance, but not that of heme *o*. Similarly, Δ*hepT* also exhibits decreased amounts of prenylated hemes compared to WT, of which only heme *a* was restored to WT-like levels upon MK-4 supplementation. Given that heme *a* synthesis is dependent on heme *o*, it is surprising that MK-4 supplementation disproportionately increases heme *a* abundance in *menE*::Tn and Δ*hepT*. The regulation of prenylated heme synthesis is not known, but these results imply that MK-4 influences the activities of CtaB and CtaA in MK-deficient strains. Notably, Δ*hepT ispA*::Tn does not produce detectable quantities of heme *o* or *a* (Fig. 4B and C), indicating that both HepT and IspA are capable of contributing to prenylated heme production. These findings demonstrate that the QoxABCD activity in the Δ*hepT ispA*::Tn double mutant is impaired due to a lack of prenylated heme cofactors.

### CydAB function is not stimulated by MK-4

**Fig 5:**
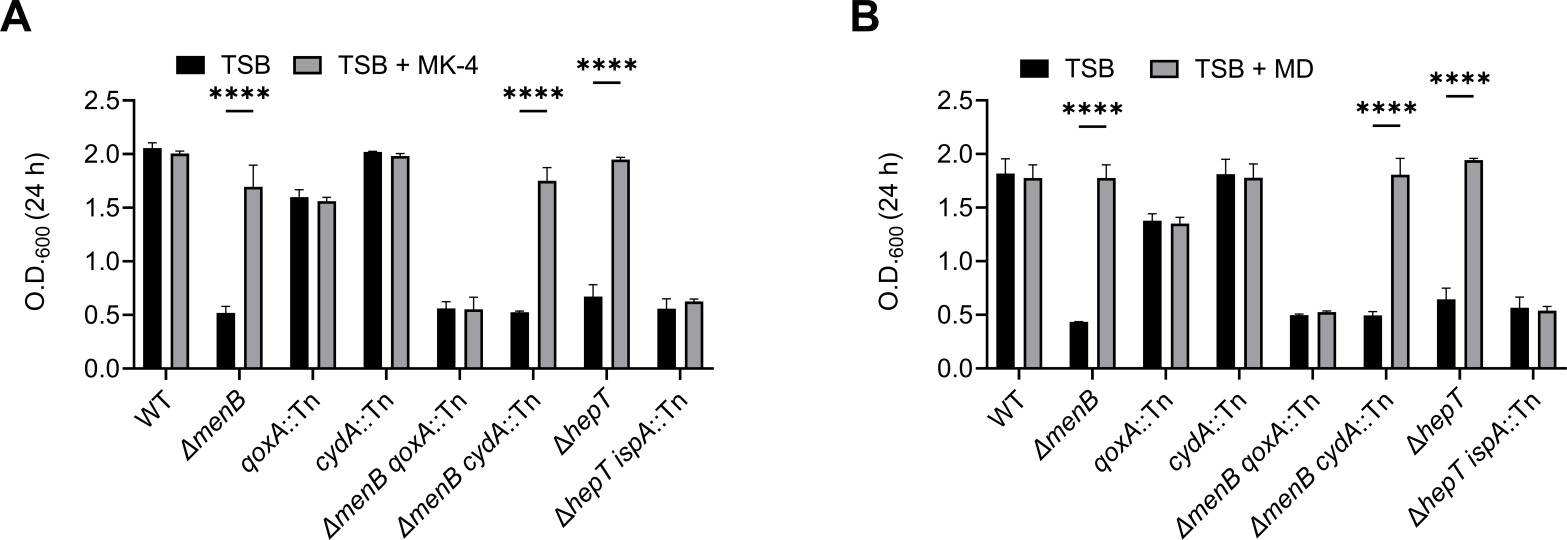
MK-4 and menadione fail to stimulate aerobic respiration in cells restricted to CydAB-dependent respiration (**A and B**). O.D._600_ values were collected after 24 h of incubation in TSB supplemented with either 12.5 µM MK-4 (**A**) or 2.5 µM menadione (MD) (**B**). Error bars represent one standard deviation from the mean. Statistical significance was determined via two-way ANOVA with Bonferroni correction. **** represents a *P*-value of <0.0001. Data are the average of three independent biological replicates performed in triplicate.

The inability to produce prenylated heme cofactors accounts for impairment of QoxABCD in Δ*hepT ispA*::Tn, but both CydAB and QoxABCD must be inactive to induce the SCV phenotype. Therefore, we reasoned that the CydAB activity is not stimulated by MK-4. We showed that *S. aureus* produces five MK species: MK-5, MK-6, MK-7, MK-8, and MK-9. Previous experiments used MK-4, an MK analog that is not natively produced by *S. aureus*, to chemically complement MK deficiency (Fig. 3A and B). To determine whether MK-4 serves as an electron carrier substrate for CydAB, a Δ*menB qoxA*::Tn double mutant was generated. In this strain, respiration is dependent on CydAB and exogenous MKs. The MK-4 supplementation of Δ*menB qoxA*::Tn did not promote significant proliferation after 24 h of incubation compared to the un-supplemented condition, which is similar to the phenotype observed for Δ*hepT ispA*::Tn (Fig. 5A). Importantly, both Δ*menB* and Δ*menB cydA*::Tn, a strain restricted to QoxABCD for aerobic respiration, exhibited increased proliferation when supplemented with MK-4 (Fig. 5A). To determine if the shortened isoprenoid affects whether MK-4 can promote the CydAB activity, the experiment was repeated using the MK analog menadione (MD), which lacks an isoprenoid moiety. While MD also increased the proliferation of Δ*menB* and Δ*menB cydA*::Tn, it failed to enhance the proliferation of Δ*menB qoxA*::Tn, mimicking the Δ*hepT ispA*::Tn phenotype (Fig. 5B). Importantly, supplementation with either MK-4 or MD increased the Δ*hepT* proliferation, indicating that the mutation of *hepT* alone is not responsible for the respiration phenotype observed in Δ*hepT ispA*::Tn. These data suggest that while Δ*hepT ispA*::Tn exhibits a defect in isoprenoid biosynthesis, its inability to perform aerobic respiration stems from the electron carrier preference of CydAB, which cannot effectively utilize MK-4. To provide further evidence of this, Δ*ispA qoxA*::Tn and Δ*hepT qoxA*::Tn were constructed. The Δ*ispA qoxA*::Tn mutant exhibited similar colony size and doubling time to the WT, indicating that respiration is intact in this strain (Fig. S5A and B). However, Δ*hepT qoxA*::Tn displayed the SCV phenotype that also fails to respond to the MK-4 supplementation, the same phenotype exhibited by Δ*hepT ispA*::Tn (Fig. S5C and D). Together, these data show that the CydAB terminal oxidase cannot utilize MK-4 or MD as electron carriers.

### PDS activity supports host colonization

**Fig 6:**
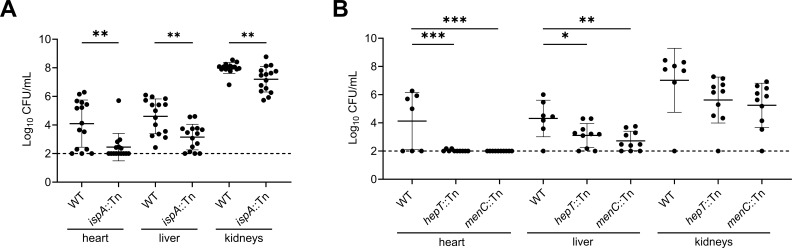
Disruption of isoprenoid synthesis impairs *S. aureus* colonization across multiple organs during systemic infection. (**A**) Bacterial burdens of WT or *ispA*::Tn in the heart, liver, and kidneys quantified after 96 h of infection and represented as colony forming units (CFU) per milliliter (CFU/mL). Statistical significance was determined via unpaired Mann-Whitney test. (**B**) Bacterial burdens of WT, *hepT*::Tn, and *menC*::Tn after 96 h of infection represented as CFU/mL. Prior to infection, *hepT*::Tn and *menC*::Tn were supplemented with 12.5 µM MK-4 to achieve a WT-like inoculum. Statistical significance was determined via one-way ANOVA with Tukey correction. (**A and B**) Error bars represent one standard deviation from the mean. *, **, and *** represent *P*-values of <0.05, <0.01, and <0.001, respectively.

Isoprenoid biosynthesis contributes to three cellular pathways: carotenoid pigment production, cell envelope synthesis, and respiration. Importantly, each of these pathways contributes to virulence or host colonization (19, 38, 53, 60, 61). Therefore, we sought to determine whether the disruption of PDS function impacts fitness during infection. The heart, liver, and kidneys of systemically infected mice were harvested 96 h post-infection, and bacterial burdens were quantified. The *ispA*::Tn mutant exhibited significantly reduced abundance in the heart, liver, and kidneys, indicating that *ispA* plays a role in host colonization (Fig. 6A). Additionally, *hepT*::Tn displayed significantly reduced burdens in the heart and liver of systemically infected mice (Fig. 6B), a phenotype that is similar to an MK-deficient *menC*::Tn mutant. The similarity between these results suggests the colonization defect observed for *hepT*::Tn could be due to a loss of MK production, but further experimentation is required to rule out the contribution of HepT to the other isoprenoid pathways.

## DISCUSSION

Addressing how bacterial isoprenoid synthesis is initiated in the absence of the short-chain PDS, IspA, is essential to understanding this pathway and its products. Our data shed light on this inquiry by demonstrating that functional redundancy sustains isoprenoid synthesis in *S. aureus*. We found that the medium-chain PDS HepT functions in three pathways: it is essential for MK synthesis but also plays a role in the lipid II cycle and supports production of prenylated hemes. While it has been hypothesized that HepT synthesizes the isoprenoid moieties used for MK synthesis (32, 37, 62), our results experimentally verify this function. We demonstrate that HepT inactivation results in the loss of MK-5, MK-6, MK-7, MK-8, and MK-9, providing direct evidence that HepT is needed for the synthesis of these MK species. Cells lacking MK exhibit a distinct colony phenotype, called the small colony variant (37, 41, 63, 64). SCV proliferation is limited due to a restricted metabolism that relies on lactic acid fermentation. Consistent with the lack of MK, the *hepT* mutant growth is impaired compared to WT, and the cells produce lactate, indicating that fermentation is the primary means by which the mutant cells generate energy. We also demonstrate the MK analog, MK-4, chemically complements proliferation and reduces lactate production. Together, these results support the conclusion that HepT is necessary for MK synthesis and respiration.

The isolation of pigmented gentamicin-resistant *ispA* mutants harboring nonsynonymous point mutations in *hepT* further supports the importance of the medium-chain PDS in aerobic respiration. Previous results demonstrated that an *ispA* mutant displays increased gentamicin resistance (27). Gentamicin is an aminoglycoside antibiotic, which relies on the PMF to enter a bacterial cell. Consequently, gentamicin resistance has a known correlation with the respiratory status of the cell (63, 65, 66). This led Krute et al. to conclude that aerobic respiration is impaired in *ispA* mutants likely due to decreased synthesis of MK and prenylated heme cofactors (27). However, the parental *ispA* mutant produces MK-8 and MK-9 and generates WT levels of lactate, demonstrating that respiration is intact in this mutant. Conversely, *ispA* mutants harboring the *hepT*^A72E^ allele exhibit significantly reduced MK pools and decreased growth kinetics. Previous work established that MK mutants are gentamicin-resistant (67). Together, these facts indicate that aerobic respiration is active in the *ispA* mutant but reduced in cells encoding the *hepT*^A72E^ allele, providing a mechanism for gentamicin resistance. Why an *ispA* mutant is more resistant to gentamicin than the WT remains a mystery. Also, it is unclear whether pigmentation is directly selected for or is an artificial consequence of the A72E substitution. Our results are consistent with previous reports that demonstrate this mutation alters the first aspartate-rich motif, increasing the similarity between HepT and short-chain PDSs like IspA (55). This change allows the cells to produce staphyloxanthin and short-chain MK-5, MK-6, and MK-7 but not the longer-chain isoprenoids needed for MK-8 or MK-9. Notably, the complete inactivation of *hepT* via transposon insertion (*hepT*::Tn) or in-frame deletion (Δ*hepT*) also leads to hyperpigmentation. SCVs display a nonpigmented phenotype when grown on solid media (64, 68), and our data demonstrate that mutating *hepT* leads to the SCV phenotype. It is interesting, then, that *hepT* mutants are hyperpigmented. This may be explained by an altered metabolite flux toward short-chain staphyloxanthin and away from medium-chain isoprenoids when *hepT* is mutated. The altered expression of genes in the staphyloxanthin and MK biosynthetic pathways could also explain the hyperpigmentation phenotype. Future investigations quantifying staphyloxanthin precursors with concomitant transcriptional quantification of genes in this pathway and MK synthesis will determine whether metabolite reallocation and altered gene expression promote the hyperpigmentation phenotype of the *hepT* mutant.

The isolation of pigmented *ispA* mutants encoding missense *hepT* mutations led us to hypothesize that HepT is also capable of condensing IPP and DMAPP to initiate isoprenoid synthesis. In keeping with this, we predicted that the simultaneous inactivation of *ispA* and *hepT* would be synthetically lethal, as isoprenoid synthesis would not be initiated in this genetic background. Additional rationale for this prediction is provided by a biochemical investigation of HepT and HepT homologs. *B. subtilis* HepT is capable of condensing IPP and DMAPP, although the reaction is inefficient (33). A similar result was described for the *E. coli* HepT homolog IspB, which is also capable of using IPP and DMAPP as substrates (29). The fact that IspB is essential in *E. coli*, *A. baumannii*, and *C. glutamicum* under standard growth conditions supports a role for IspB beyond quinone synthesis (35, 46–48). HepT is also a requirement for *B. subtilis* viability under standard laboratory conditions, but this species can tolerate loss of HepT activity when cultured in medium that supports proliferation of L-form cells that lack peptidoglycan (49). This finding implies that HepT also supports peptidoglycan synthesis. Our results demonstrate that *S. aureus* is amenable to genetic inactivation of *hepT* in the presence or absence of *ispA*. However, Δ*hepT ispA*::Tn lacked pigmentation and did not respond to chemical complementation with the MK analog, MK-4. Subsequent experimentation demonstrated that Δ*hepT ispA*::Tn is impaired for aerobic respiration due to inactivity of both terminal oxidases, CydAB and QoxABCD. Terminal oxidases accept electrons from the quinone pool and reduce oxygen, thereby performing the last step of aerobic respiration. Our data show that QoxACBD impairment can be attributed to loss of prenylated heme cofactors, while the inability of MK-4 to stimulate respiration in Δ*hepT ispA*::Tn is dependent on CydAB. The finding that MK-4 fails to chemically complement Δ*menB qoxA*::Tn conclusively shows that CydAB is restricted to the use of long-chain MK, presumably MK-7 or greater. The functional role of short-chain MKs (MK-5 and MK-6) in supporting respiration is unclear given these MK species are produced at significantly reduced quantities compared to the longer-chain MKs (MK-7, MK-8, and MK-9). However, given the apparent preference of terminal oxidases for the isoprenoid chain length of MKs, further investigation should be carried out to precisely define correlations between MK tail length, CydAB terminal oxidase activity, and the composition of the MK pool on cellular respiration.

Investigating Δ*hepT ispA*:Tn in the context of the single mutants demonstrated that HepT uniquely contributes to MK production, while the precursor for staphyloxanthin is provided exclusively by IspA. Interestingly, either enzyme can contribute to prenylated heme production. Both single mutants exhibit decreased prenylated heme cofactors compared to WT, but levels of heme *o* in the *hepT* mutant suggest a preference for HepT. The finding that MK-4 supplementation increases heme *a* levels but not those of heme *o* in the Δ*hepT* and *menE*::Tn mutants suggests that MK influences the final product of heme prenylation. The synthesis of heme *a* is dependent on heme *o*; therefore, an abundance of heme *a* indicates that flux to heme *a* is increased in MK-4-supplemented Δ*hepT* and *menE*::Tn mutant cultures. Whether the activities of CtaA, CtaB, and CtaM are influenced by MK is currently unknown. Also, QoxABCD can be populated with either heme *o* or *a*, and how each heme cofactor might affect interactions with MK species has not been elucidated (51). Addressing these questions will define mechanisms that control heme prenylation and provide additional insights into how respiration is modulated in *S. aureus*. These results reveal that HepT can function beyond MK synthesis. Consistent with this, we monitored levels of the lipid II precursors Und-P and Und-OH, which revealed decreased abundance of both metabolites in the *hepT* mutant. Though Und-OH does not participate directly in the lipid II cycle (14), previous reports support a model whereby it serves as a reservoir for rapid conversion to Und-P via undecaprenol kinase when higher levels of Und-P are needed (69). In the *hepT* mutant, a depleted Und-OH reservoir implies the cell is attempting to maintain Und-P levels and demonstrates that HepT plays a role in maintaining Und-P. Overall, the finding that Δ*hepT ispA*::Tn is viable provides strong evidence that another enzyme is capable of condensing IPP and DMAPP to produce the FPP needed for essential lipid II. Based on this supposition and our results, a model can be considered whereby IspA, HepT, and the unknown PDS each produce FPP that is restricted to generate staphyloxanthin, MK, and lipid II, respectively (Fig. S6). However, our findings cannot rule out the possibility that FPP generated by the uncharacterized PDS can also be used as a HepT substrate. The fact that HepT homologs are capable of condensing IPP and DMAPP *in vitro*, albeit at a limited rate, implies that *S. aureus* HepT is a viable source of FPP (29, 33). In fact, Krute et al. also hypothesized that HepT, UppS, or both can produce FPP to support the growth of *ispA* mutant cells (27). However, studies of UppS substrate specificity in other bacterial species show that DMAPP is not used as a substrate by this enzyme (9, 70). Discerning between these possibilities requires further biochemical characterization of purified recombinant *S. aureus* HepT and UppS to establish substrate specificities and reaction kinetics. Nonetheless, our work demonstrates that in addition to its importance in producing MK, HepT plays a multifunctional role by contributing to the isoprenoid-dependent heme cofactor and lipid II precursor pathways.

Staphyloxanthin, MK, terminal oxidase activity, and aerobic respiration have been shown to be critical factors for *S. aureus* pathogenesis (19, 38, 53, 71). To determine the contributions of IspA and HepT to *S. aureus* host colonization, we used the systemic mouse model of infection. This analysis showed that the *hepT* mutant exhibited colonization defects in the heart and liver, which was similar to the MK-deficient *menC* mutant. This finding suggests that the loss of MK is a factor in the colonization defects displayed by the *hepT* mutant. However, further investigation is required to determine whether perturbations of the other HepT-associated isoprenoids also contribute to the decreased virulence of the mutant. The *ispA* mutant showed broader colonization defects, with reduced bacterial burden not only in the heart and liver but also in the kidneys. The disruption of isoprenoid biosynthesis has been previously established to reduce virulence, as mice treated with a chemical inhibitor of UppS exhibit increased survival compared to untreated mice in a model of systemic infection (72). Our data demonstrate that inhibiting isoprenoid synthesis at an earlier step in this pathway is an alternative strategy for reducing *S. aureus* infection.

*S. aureus* is a common nosocomial pathogen that is frequently resistant to antibiotics, presenting a clinical challenge (73). Furthermore, the presence of antibiotic-resistant *S. aureus* in hospital settings can complicate surgical recovery and lead to increased mortality (74). By identifying biological pathways that support survival in the host, we reveal potential targets that could be used for drug development and therapeutic intervention. In this study, we showed that the genetic disruption of isoprenoid biosynthesis impacted host colonization and impeded several downstream pathways that are known to support pathogenesis, revealing the potential of this pathway as a drug target. Additionally, our data support a redundant model of isoprenoid biosynthesis and showed that a high level of disruption in this pathway is tolerated by *S. aureus* but limits the pathogen’s metabolic versatility. As isoprenoid biosynthesis is a highly conserved biological process that is active in other bacterial pathogens, developing strategies for targeting bacterial isoprenoid biosynthesis may impact treatment of a wide range of microbial pathogens.

## MATERIALS AND METHODS

### Bacterial strains, plasmids, and culture conditions

*S. aureus* strain JE2 was used as the WT strain in this study. All *S. aureus* mutants were generated in the JE2 WT background as listed in Table 1. Transposon mutagenesis was carried out by propagating φ85 on the appropriate strain from the Nebraska Transposon Mutant Library and transduced into a recipient strain as described previously (20, 75). However, for generating the Δ*hepT ispA*::Tn double mutant, we found no colonies were obtained when plating on TSA (Remel) supplemented with 10 µg/mL erythromycin and 40 mM sodium citrate. Instead, plating on TSA supplemented with 10 µg/mL erythromycin without sodium citrate yielded colonies. In-frame deletions were generated via an allelic exchange protocol with the pKOR1mcs-Δ*hepT* plasmid as described previously and confirmed via PCR (76, 77). The previously generated pKOR1-Δ*menB* plasmid was used to generate the Δ*menB* deletion mutant in the JE2 background (37). Cloning and PCR confirmation of mutants were performed using the primers listed in Table S2. All overnight cultures were started from single colonies in 5 mL TSB (Fisher Scientific) and incubated overnight at 37°C at 225 rpm unless stated otherwise. Mutants deficient for MK synthesis were supplemented with 12.5 µM MK-4 in overnight cultures. All strains harboring the pOS1 plasmid were grown in the presence of 10 µg/mL chloramphenicol. Genes were cloned into pOS1 via restriction ligation or Gibson assembly, as denoted in Table S2, transformed into *E. coli* DH5α, and passaged through the restriction minus modification plus *S. aureus* strain RN4220 prior to being transformed into the respective JE2 strain.

**TABLE 1:** Strains used in this study

Strain	Description	Reference
RN4220	Restriction-deficient, methylation-proficient cloning intermediate	(78)
JE2	USA300, wild type (WT)	(20)
*ispA*::Tn	*ispA* transposon from NE1447 backcrossed into WT JE2	This study
*hepT*::Tn	*hepT* transposon from NE1920 backcrossed into WT JE2	This study
Δ*hepT*	In-frame deletion of *hepT* constructed using pKOR1-Δ*hepT*	This study
Δ*hepT ispA*::Tn	*ispA* transposon from NE1447 transduced into Δ*hepT*	This study
*menE*::Tn	Tn917 inserted into *menE* backcrossed into WT JE2	(71)
Δ*menB*	In-frame deletion of *menB* constructed using pKOR1-Δ*menB*	This study
*menC*::Tn	Tn917 inserted into *menC* backcrossed into WT JE2	(71)
*qoxA*::Tn	*qoxA* transposon from NE92 backcrossed into WT JE2	This study
*cydA*::Tn	*cydA* transposon from NE117 backcrossed into WT JE2	This study
Δ*menB qoxA*::Tn	*qoxA* transposon from NE92 transduced into Δ*menB*	This study
Δ*menB cydA*::Tn	*cydA* transposon from NE117 transduced into Δ*menB*	This study
*crtM*::Tn	*crtM* transposon from NE2499 backcrossed into WT JE2	This study
Δ*hepT crtM*::Tn	*crtM* transposon from NE2499 transduced into Δ*hepT*	This study
Δ*ispA*	In-frame deletion of *ispA* constructed using pKOR1-Δ*ispA*	This study
Δ*ispA qoxA*::Tn	*qoxA* transposon from NE92 transduced into Δ*ispA*	This study
Δ*ispA cydA*::Tn	*cydA* transposon from NE117 transduced into Δ*ispA*	This study
Δ*hepT qoxA*::Tn	*qoxA* transposon from NE92 transduced into Δ*hepT*	This study
Δ*hepT cydA*::Tn	*cydA* transposon from NE117 transduced into JE2 Δ*hepT*	This study
*ispA*::Tn suppressor 115	Gentamicin-resistant pigmented *ispA*::Tn isolate that harbors the HepT^A72E^ allele	This study
*ispA*::Tn suppressor 144	Gentamicin-resistant pigmented *ispA*::Tn isolate that harbors the HepT^A72E^ allele	This study
*ispA*::Tn suppressor 164	Gentamicin-resistant pigmented *ispA*::Tn isolate that harbors the HepT^A72E^ allele	This study
*ispA*::Tn suppressor 165	Gentamicin-resistant nonpigmented *ispA*::Tn isolate that harbors the HepT^A165T^ allele	This study
JE2 pOS1	Empty pOS1 plasmid vector	This study
*ispA*::Tn pOS1	Empty pOS1 plasmid vector	This study
*ispA*::Tn pOS1-*ispA*	*ispA*::Tn harboring a pOS1 plasmid encoding the *ispA* gene under the control of the P*_lgt_* promoter	This study
Δ*hepT* pOS1	Empty pOS1 plasmid vector control	This study
Δ*hepT* pOS1-*hepT*	Δ*hepT* harboring a pOS1 plasmid encoding the *hepT* gene under the control of the P*_lgt_* promoter	This study
Δ*hepT ispA*::Tn pOS1	Empty pOS1 plasmid vector control	This study
Δ*hepT ispA*::Tn pOS1-*hepT*	Δ*hepT ispA*::Tn harboring a pOS1 plasmid encoding the *hepT* gene under the control of the P*_lgt_* promoter	This study
Δ*hepT ispA*::Tn pOS1-*ispA*	Δ*hepT ispA*::Tn harboring a pOS1 plasmid encoding *ispA* under the control of the P*_lgt_* promoter	This study
Δ*hepT ispA*::Tn pOS1-*hepT*^A72E^	Δ*hepT ispA*::Tn harboring a pOS1 plasmid encoding the *hepT*^A72E^ allele from *ispA*::Tn suppressor 115 under the control of the P*_lgt_* promoter	This study

### Isolation of gentamicin-resistant pigmented *ispA*::Tn suppressor mutants

An overnight culture of *ispA*::Tn was prepared from a single colony in TSB. A 1:10 dilution of the 10^9^ CFU/mL overnight culture was made and spread onto a TSA plate supplemented with 3 µg/mL gentamicin. The plate was incubated at 37°C, and pigmented colonies were observed after ~72 h of incubation. Pigmented colonies were isolated on TSA, and a single colony was used to make an overnight culture to ensure a clonal population was obtained. The overnight cultures were used to make frozen archival stocks, and cells used for subsequent experimentation involving the gentamicin-resistant pigmented *ispA*::Tn suppressors were recovered from these stocks. Table S1 lists single nucleotide polymorphisms (SNPs) present in each suppressor isolate.

### Whole-genome sequencing and analysis

Genomic DNA was extracted from 1 mL of the overnight cultures using a Wizard Genomic DNA Purification Kit (Promega). Genomic DNA was sequenced via Illumina sequencing at the Duke University Sequencing and Genomic Technologies core facility. Genomic analyses, including paired-end read trimming, mapping, and SNP calling, were performed using Geneious Prime version 2024.0.5. The genome of *S. aureus* strain USA300_FPR3757 (GenBank accession number: CP000255.1) was used as a reference genome to which sequencing data were mapped.

### Quantification of isoprenoid-derived metabolites via high-performance liquid chromatography mass spectrometry

MKs and C_55_ isoprenoids were extracted and analyzed as described previously with slight modifications (29). *S. aureus* cultures were grown in 50 mL of TSB at 37°C with shaking at 225 rpm until stationary phase. Cultures were pelleted and resuspended in 2 mL of methanol:0.3% NaCl (10:1, v/v). Vitamin K_1_, solanesol, and solanesyl phosphate were added as internal standards. To extract MKs and Und-OH, hexane was added to the cell suspension and vortexed, and the upper phase was collected. The remaining Und-OH and Und-P in the aqueous layer were treated with alkali by adding 1 mL 60% KOH and boiled for 60 min. Diethyl ether was added and vortexed, then collected after phase separation. The collected diethyl ether was washed with 5% acetic acid.

The hexane solution containing MKs and polyprenols was loaded onto a 0.4 g column of neutral alumina (grade III). MKs were eluted with 2.4% diethyl ether in hexane, and polyprenols were eluted with 10% diethyl ether in hexane. MKs and polyprenols were run on an LCMS-2010 (Shimadzu Co.) using an STR ODS_II column (Shinwa Chemical Ind. Ltd.). A 2-propanol:methanol (1:1, v/v) mixture was used as the mobile phase at a flow rate of 0.1 mL/min. The diethyl ether extract was divided in half. One half was dried under nitrogen gas, and the remaining residue was resuspended in hexane. The hexane solution was loaded onto a neutral alumina column, and polyprenols were eluted with 10% diethyl ether in hexane as described above. The other half of the diethyl ether extract was dried under nitrogen gas, and the remaining residue was resuspended in chloroform:methanol (2:1, v/v). The chloroform:methanol (2:1, v/v) solution containing polyprenyl phosphates derived by alkaline hydrolysis was loaded onto an ion exchange cartridge (Supelclean LC-NH_2_). Polyprenyl phosphates were eluted with a chloroform:methanol:water (2:0.9:0.1, v/v/v) solution containing 0.1 M ammonium acetate and analyzed via HPLC using a STR ODS_II column with 2-propanol:methanol (1:1, v/v) containing 5 mM phosphoric acid.

### Growth curve analysis and end-point optical density reading

Overnight cultures were pelleted and resuspended in 137 mM NaCl, 2.7 mM KCl, 10.1 mM Na_2_HO_4_P, and 1.8 mM KH_2_O_4_P phosphate-buffered saline pH 7.4 (PBS) and placed on ice. The optical density at 600 nm (O.D._600_) was measured for resuspended cultures and normalized to an O.D._600_ equal to 1.0 (or ~3.5 × 10^9^ CFU/mL). A volume of 150 µL growth medium was dispensed into each well of a 96-well plate, and 1.5 µL of the normalized culture (1:100 dilution) was used to inoculate each well. The plate was incubated in a Stratus (Cerillo) plate reader at 37°C with shaking at 300 rpm. To calculate the doubling time, the following equation was used: doubling time = (Ln(O.D._2_) – Ln(O.D._1_)) / (T_2_ – T_1_), where O.D._1_ represents the first O.D._600_ reading at the start of the mid-log phase, and O.D._2_ represents the last O.D._600_ reading of the mid-log phase. T_1_ and T_2_ represent the time points at which O.D._1_ and O.D._2_ were collected, respectively. For growth analysis where the end-point, stationary-phase O.D._600_ was collected, a 96-well plate was prepared and incubated as described above. After the designated incubation time, the plate was removed from the Stratus plate reader, and each well was carefully pipetted up and down to ensure all cells were resuspended. A single time point was then collected by measuring the O.D._600_ using an H1 Synergy (Biotek) plate reader.

### Measuring l-lactate production

The EnzyChrom l-Lactate Assay Kit (ECLC-100, BioAssay Systems) was used to assess the l-lactate production in *S. aureus* cultures. Overnight cultures were pelleted and normalized to an O.D._600_ of 1.0 in PBS and placed on ice. Normalized cultures were subcultured 1:100 in 5 mL fresh TSB and incubated at 37°C with shaking at 225 rpm for 15 h. Cultures were then pelleted, and the supernatant was collected, sterile-filtered, and stored at −20°C. Sterile supernatants were diluted 1:10 in sterile Milli-Q water, and 20 µL was dispensed into a well of a 96-well plate. The reaction buffer was prepared by mixing the following reagents from the l-lactate assay kit: 60 µL assay buffer, 1 µL enzyme A, 1 µL enzyme B, 10 µL NAD, and 14 µL MTT. In a 96-well plate, 20 µL of the diluted supernatant was added to a well for each sample. An 80 µL volume of reaction buffer was added to each well and mixed briefly by pipetting up and down. The initial O.D._565_ was measured immediately after mixing using an H1 Synergy plate reader, and then the plate was incubated at room temperature for 20 min. The O.D._565_ was measured again, and the initial O.D._565_ measurement was subtracted from the second measurement to yield the sample values. The sample values were compared to a standard curve (prepared as described in the l-lactate assay kit) to determine l-lactate concentrations.

### Heme quantification via HPLC

In a 125 mL Erlenmeyer flask, 20 mL of TSB was inoculated with a single colony and grown overnight at 37°C with shaking at 225 rpm. Cultures were pelleted and resuspended in 200 µL molecular biology grade water (Cytiva: SH30538.03). A volume of 100 µL of the cell suspension was transferred to a 1.5 mL Eppendorf tube. An acid:acetone solution was prepared, consisting of five parts 12 M HCl to 95 parts acetone. A 150 µL volume of acid acetone was added to the cell suspension and mixed briefly by vortexing. The mixture was incubated on ice for 10 min and vortexed for 20 s. The mixture was further incubated for 10 min and then vortexed for 20 s. The cells were pelleted, and the supernatant was collected. The supernatant was centrifuged again, and the resulting supernatant was transferred to an HPLC injection vial.

Hemes were analyzed with the Agilent 1260 Infinity HPLC System using the InfinityLab Poroshell EC-C18 reverse-phase column (Agilent: 699975-902) coupled with a diode array detector (Agilent: G1315D). A 10 µL volume of sample was injected, and hemes were separated using a gradient of solvents A (0.1% v/v trifluoroacetic acid in Milli-Q water) and B (0.1% v/v trifluoroacetic acid in acetonitrile). The gradient progressed as follows: 25% solvent B (0.00–2.67 min), 25–55% solvent B (2.67–4.33 min), 55–75% solvent B (4.33–11.00 min), 75–100% solvent B (11.00–12.67 min), 100% solvent B (12.67–20.00 min), and a gradient returning to 25% solvent B (20.00–23.00 min). Using this method, heme *b* elutes at 6.8 min, heme *a* at 10.8 min, and heme *o* at 12 min. Hemes were detected by measuring absorbance at 400 nm and identified by comparing retention time and the absorbance maximum (79). HPLC chromatograms were used to determine the area under the peak for each of the heme species. Normalized heme abundance was determined by dividing the peak area of hemes *o* and *a* by the peak area of heme *b*.

### Systemic mouse infections

Overnight cultures were subcultured 1:100 in 5 mL fresh TSB and incubated at 37°C with shaking at 225 rpm for 3 h. MK-4 was used to supplement *menC*::Tn and *hepT*::Tn cultures to induce respiration and promote growth to WT and *ispA*::Tn mutant levels. Cultures were pelleted at 4°C and washed once in 12 mL of Dulbecco’s PBS (DPBS, Sigma-Aldrich: D8537). Resuspended cultures were pelleted again and normalized to an O.D._600_ of 0.4 in DPBS and kept on ice. Eight-week-old female BALB/c mice (Jackson Laboratories) were retro-orbitally infected with 10^7^ CFU of the indicated *S. aureus* strain. Mice were euthanized via CO_2_ inhalation 96 h post-infection, and the heart, liver, and kidneys were harvested. The heart and kidneys were homogenized via bead beating (1.5 mL RINO lysis beads, NextAdvance, Inc.) in 500 μL DPBS using Bullet Blender Storm24 (NextAdvance, Inc.). Once homogenized, 500 μL DPBS was added to the lysate. The livers were manually homogenized in a Whirl-pak (Nasco) containing 1 mL DPBS. Organ homogenates were serially diluted in DPBS and plated on TSA for enumeration. All infections were performed at Michigan State University under the principles and guidelines described in the *Guide for the Care and Use of Laboratory Animals* described in protocol PROTO202200474, which received approval from the Michigan State University Institutional Animal Care and Use Committee.

## SUPPLEMENTAL MATERIAL

Supplemental figures and tables mbio.00353-25-s0001.docx Figure S1 to S6 and Table S1 and S2.

ASM does not own the copyrights to Supplemental Material that may be linked to, or accessed through, an article. The authors have granted ASM a non-exclusive, world-wide license to publish the Supplemental Material files. Please contact the corresponding author directly for reuse.
