# Supplementary material for: A redundant isoprenoid biosynthetic pathway supports Staphylococcus aureus metabolic versatility
Source: mBio. 2025 Jun 30;16(8):e00353-25. doi: 10.1128/mbio.00353-25 (PMC12345219; doi:10.1128/mbio.00353-25)
Supplement: Supplemental figures and tables — Figure S1 to S6 and Table S1 and S2. [file mbio.00353-25-s0001.docx]

| **Table S1: In-frame mutations in *ispA*::Tn gentamicin resistant colonies** | | | | | | |
| --- | --- | --- | --- | --- | --- | --- |
| **Strain** | **Nucleotide** | **Locus Tag** | **Gene** | **Mutation** | **Protein Effect** | **Polymorphism** |
| *ispA*::Tn^115^ |  |  |  | |  |  |
|  | 1286986 | SAUSA300_1169 | *ftsK* | A -> G | K180R | Transition |
|  | 1527020 | SAUSA300_1359 | *hepT* | G -> T | A72E | Transversion |
|  | 1765020 | SAUSA300_1611 | *valS* | A -> C | None | Transversion |
|  | 1939443 | SAUSA300_1753 | *splF* | C -> G | G11A | Transversion |
|  | 2150262 | SAUSA300_1993 | *fruC* | (C)4 -> (C)5 | Frame Shift | Insertion |
| *ispA*::Tn^144^ |  |  |  |  |  |  |
|  | 1286986 | SAUSA300_1169 | *ftsK* | A -> G | K180R | Transition |
|  | 1527020 | SAUSA300_1359 | *hepT* | G -> T | A72E | Transversion |
|  | 1765020 | SAUSA300_1611 | *valS* | A -> C | None | Transversion |
|  | 1939443 | SAUSA300_1753 | *splF* | C -> G | G11A | Transversion |
|  | 2150262 | SAUSA300_1993 | *fruC* | (C)4 -> (C)5 | Frame Shift | Insertion |
| *ispA*::Tn^164^ |  |  |  |  |  |  |
|  | 1527020 | SAUSA300_1359 | *hepT* | G -> T | A72E | Transversion |
|  | 1765020 | SAUSA300_1611 | *valS* | A -> C | None | Transversion |
|  | 1939443 | SAUSA300_1753 | *splF* | C -> G | G11A | Transversion |
| *ispA*::Tn^165^ |  |  |  |  |  |  |
|  | 1286986 | SAUSA300_1169 | *ftsK* | A -> G | K180R | Transition |
|  | 1526742 | SAUSA300_1359 | *hepT* | C -> T | A165T | Transition |
|  | 1765020 | SAUSA300_1611 | *valS* | A -> C | None | Transversion |
|  | 1939443 | SAUSA300_1753 | *splF* | C -> G | G11A | Transversion |
|  | 2150262 | SAUSA300_1993 | *fruC* | (C)4 -> (C)5 | Frame Shift | Insertion |
|  | | | | | | |

| **Table S2: List of primers** | | |
| --- | --- | --- |
| **Primer name** | **Sequence (5'-3')** | **Reference** |
| **Transposon confirmation primers**: |  |  |
| *ispA*::Tn_Check | AGAAACGCAAAGTTTTGAAGAAA | This study |
| *hepT*::Tn_Check | TGCAATGCACCTTGGCTAT | This study |
| *cydA*::Tn_Check | GCGTGATATTTCTCTCTTCAAAATCAA | This study |
| *qoxA*::Tn_Check | CATATTTTCTTCACTAGTGAAGTTTGGATC | This study |
| *crtM*::Tn_Check | TACTGCAATCTTCATTATTCAACCACC | This study |
| NE_Buster | GCTTTTTCTAAATGTTTTTTAAGTAAATCAAGTACC | (20) |
| NE_Martn | AAACTGATTTTTAGTAAACAGTTGACGATATTC | (20) |
| **Deletion confirmation primers**: |  |  |
| *hepT*_Del_Check_F | GGTATCTCATACACACTCGCTCCTTTC | This study |
| *hepT*_Del_Check_R | GTGATAATATCGTGAGGTGTAGACATGGA | This study |
| *ispA_*Del_Check_F | CAACAAAGACTGCGTTTCATGTTGG | This study |
| *ispA_*Del_Check_R | CGTTATAAGTGCCATGATGTTCAAAGGTAG | This study |
| *menB*_Del_Check_F | AAAAATCAATTTGTATACGTCATG | This study |
| *menB*_Del_Check_R | GGTCACATCCCTATATCTAATTTG | This study |
| **Gibson assembly primers**: |  |  |
| pKOR1_*hepT*_up_F | TTCATAAATAGTTTAACTTTGCCACGTTAATC | This study |
| pKOR1_*hepT*_up_R | CGGAACCGGTACCAATGGATATTGAAATCTTCATTACATCATC | This study |
| pKOR1_*hepT*_d_F | GCTGCTAGCTAGCTAGAGATAAAGTTAATCAGTCCGTTTAAAAAAATTATG | This study |
| pKOR1_*hepT*_d_R | CAAAGTTAAACTATTTATGAAAAGTATTGAAAGCG | This study |
| pKOR1_*ispA*_up_F | GCTGCTAGCTAGCTAGAGATTTATTCATCGGTAGATTCG | This study |
| pKOR1_*ispA*_up_R | CGGGTTCAGGTATGCAGATAGTTTAGGTGTAG | This study |
| pKOR1_*ispA*_d_F | TATCTGCATACCTGAACCCGTTTCACCAC | This study |
| pKOR1_*ispA*_d_R | CGGAACCGGTACCAATGGATAAGCAAATATATCGATTAGCAACAATTG | This study |
| pOS1_*hepT*_F | TGAACATATGCTCGAGGATCATGAACAATGAAATTAAGAAAGTGGAACA | This study |
| pOS1_*hepT*_R | AGCTTGGCTGCAGGTCGACGCTACGTGTTTCTTGAACCCAT | This study |
| **Restriction enzyme cloning primers**: |  |  |
| pOS1_*ispA*_F | GCGCATATGACGAATCTACCGATGAATAAATT | This study |
| pOS1_*ispA*_R | GCGGGATCCTTAGTGATCCCTGCTATAAAATA | This study |
|  | | |


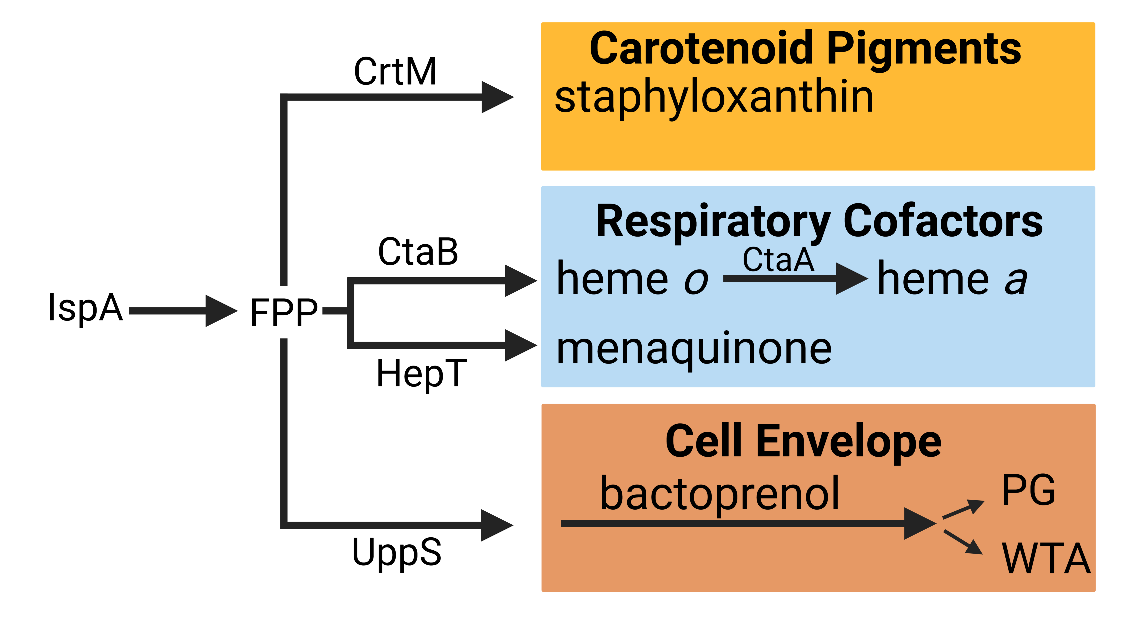


**Figure S1: An illustration of isoprenoid synthesis in *Staphylococcus aureus*.**

The previous model of isoprenoid synthesis in *S. aureus* showing that farnesyl diphosphate (FPP) is exclusively produced by IspA. FPP is used by four enzymes to support three cellular pathways: carotenoid pigment production, respiratory cofactor synthesis, and cell envelope maintenance. CrtM condenses two FPPs to produce the carotenoid pigment staphyloxanthin. CtaB adds FPP to heme *b*, prenylating the heme group and generating heme *o*, which is subsequently converted to heme *a* by CtaA. HepT elongates FPP for use in menaquinone synthesis. UppS elongates FPP for production of the C_55_ isoprenoid bactoprenol, which is essential for the synthesis of peptidoglycan (PG) and wall teichoic acid (WTA).


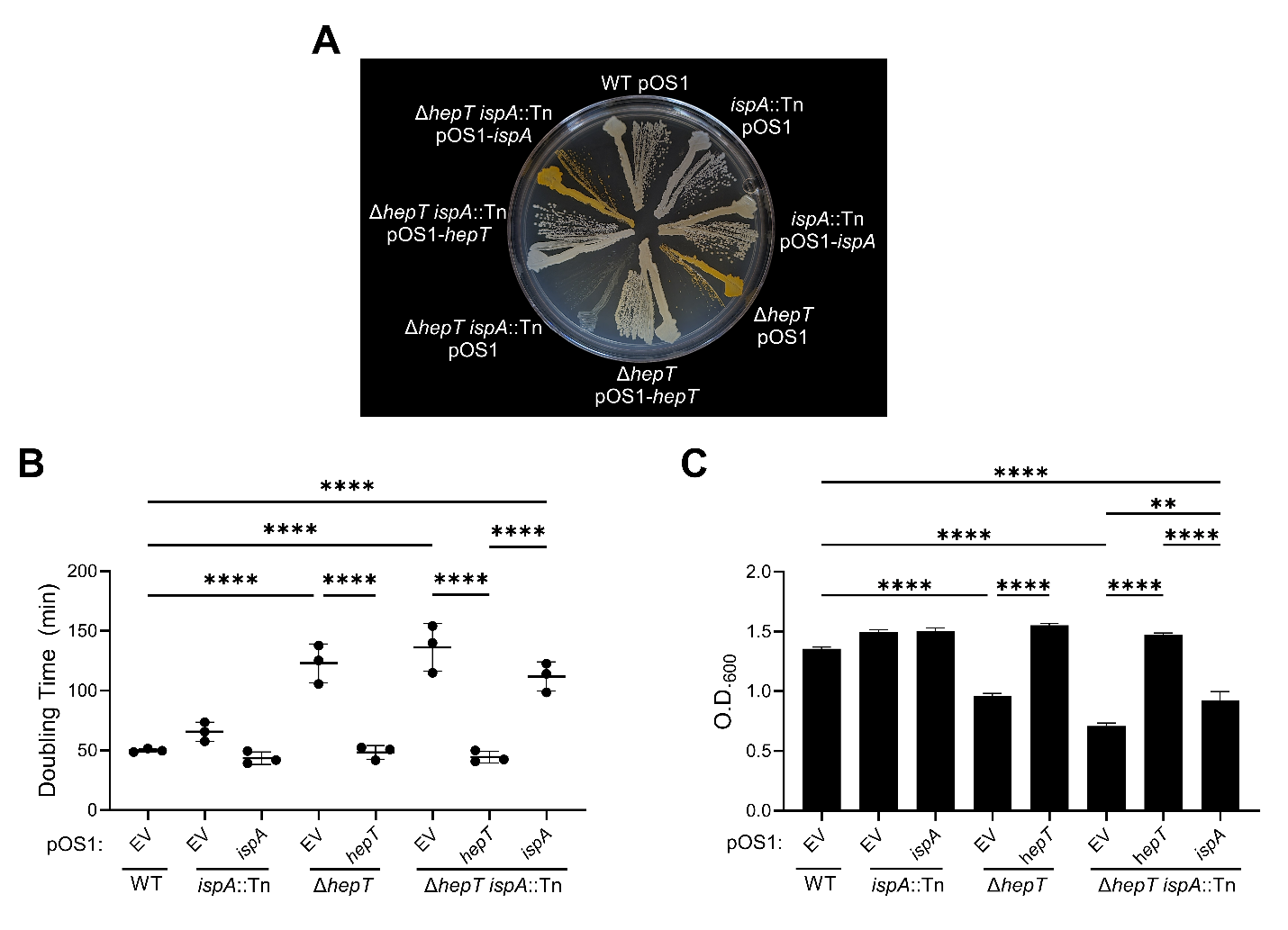


**Figure S2: ∆*hepT ispA*::Tn harboring plasmid encoded copies of *hepT* or *ispA* exhibit phenotypes that correspond to the respective single mutants.**

**A.** Overnight cultures of the indicated strains were streaked onto TSA supplemented with 10 µg/mL chloramphenicol and 12.5 µM MK-4. **B.** Growth curve analysis of the indicated strains cultured in TSB supplemented with 10 µg/mL chloramphenicol. Data are the average of three independent biological replicates performed in technical triplicate. Error bars represent one standard deviation from the mean. **C.** Stationary phase O.D._600_ collected at 12 hours of incubation. Data are the average of three independent biological replicates performed in technical triplicate. Error bars represent one standard deviation from the mean. Statistical significance in panels B and C was determined via one-way ANOVA. ** and **** represent p-values of <0.01 and 0.0001, respectively.


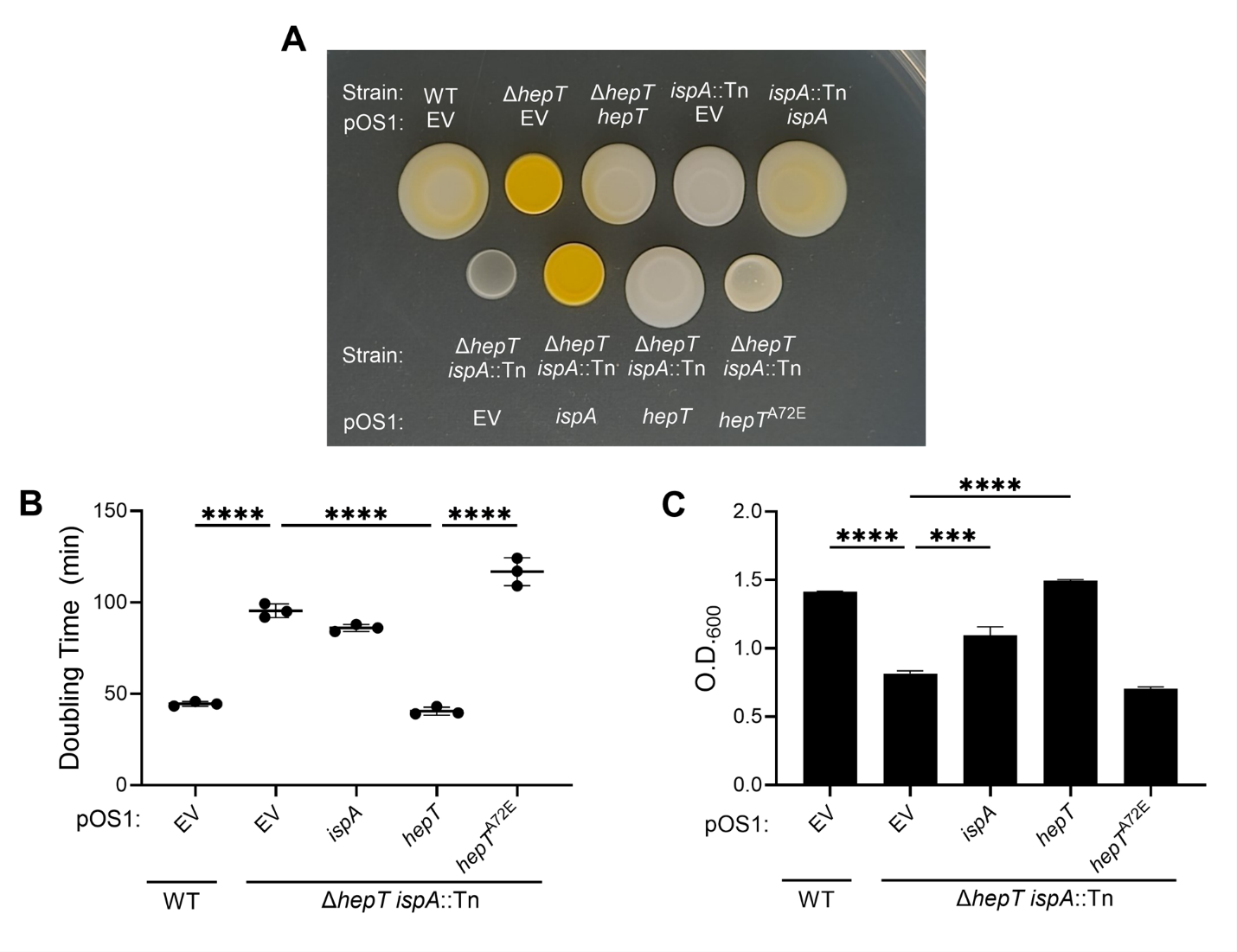


**Figure S3: The *hepT*^A72E^ allele restores pigmentation to Δ*hepT ispA*::Tn but fails to complement growth.**

**A.** Colonies generated from 5 µL of overnight cultures of the indicated strain spotted onto TSA supplemented with 10 µg/mL chloramphenicol and imaged after 72 hours of incubation. **B.** Doubling times of the indicated strains represented in minutes (min). **C.** Stationary phase O.D._600_ of the indicated strains after 12 hours of incubation. In panels B and C error bars represent one standard deviation from the mean. Statistical significance was determined via one-way ANOVA. *** and **** represent p-values of <0.001 and <0.0001, respectively.


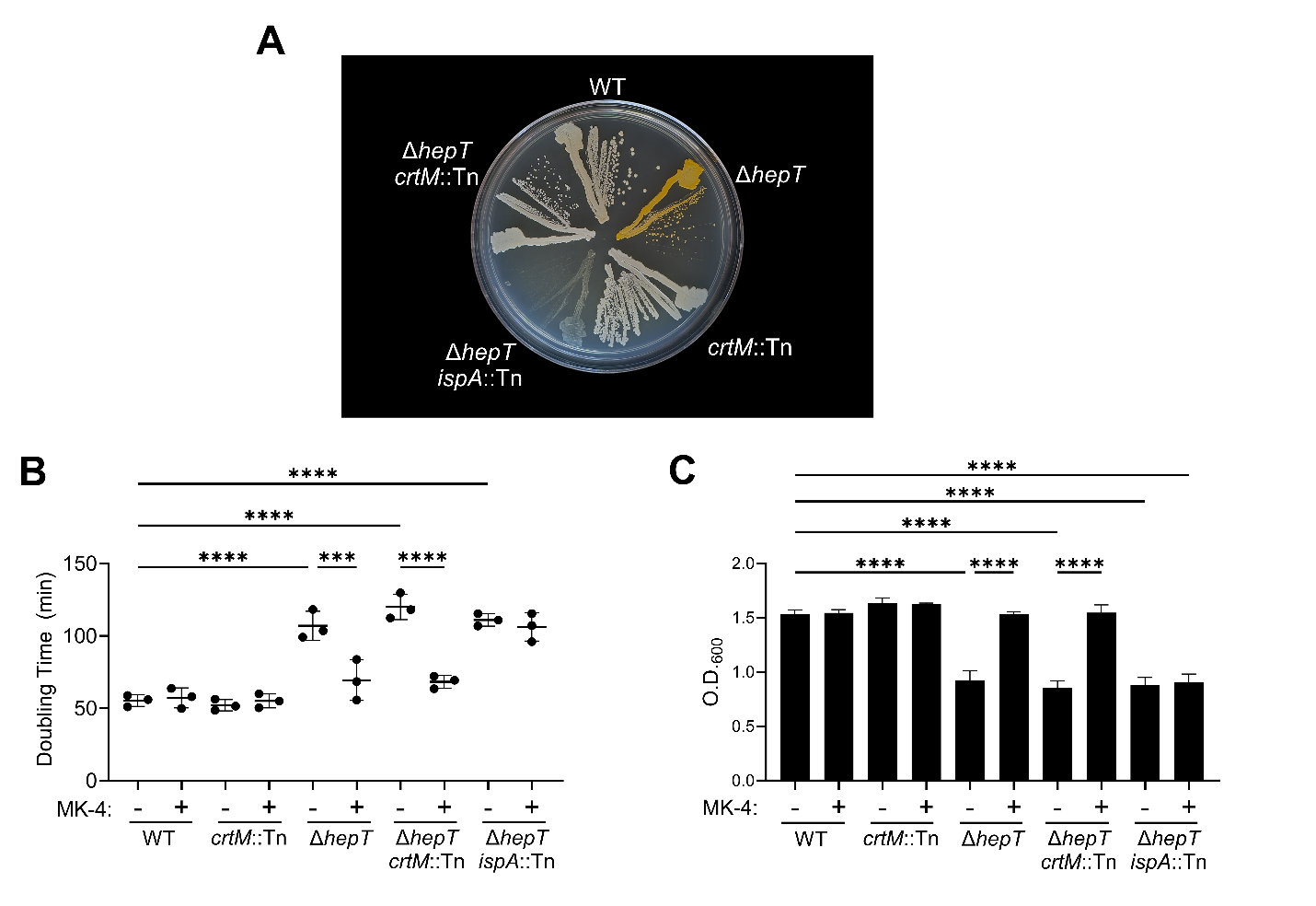


**Figure S4: Loss of pigmentation does not induce the small colony variant phenotype or prevent MK-4 chemical complementation of Δ*hepT*.**

**A.** The indicated strains streaked for isolation on TSA supplemented with 12.5 µM MK-4 and incubated overnight. **B.** Doubling times of the indicated strains cultured in TSB with or without supplementation with 12.5 µM MK-4. **C.** Stationary phase O.D._600_ of the indicated strains after 12 hours of incubation. Data presented in B and C are the average of three independent biological replicates performed in technical triplicate. Error bars represent one standard deviation from the mean. Statistical significance was determined via one-way ANOVA. *** and **** represent p-values of <0.001 and <0.0001, respectively.


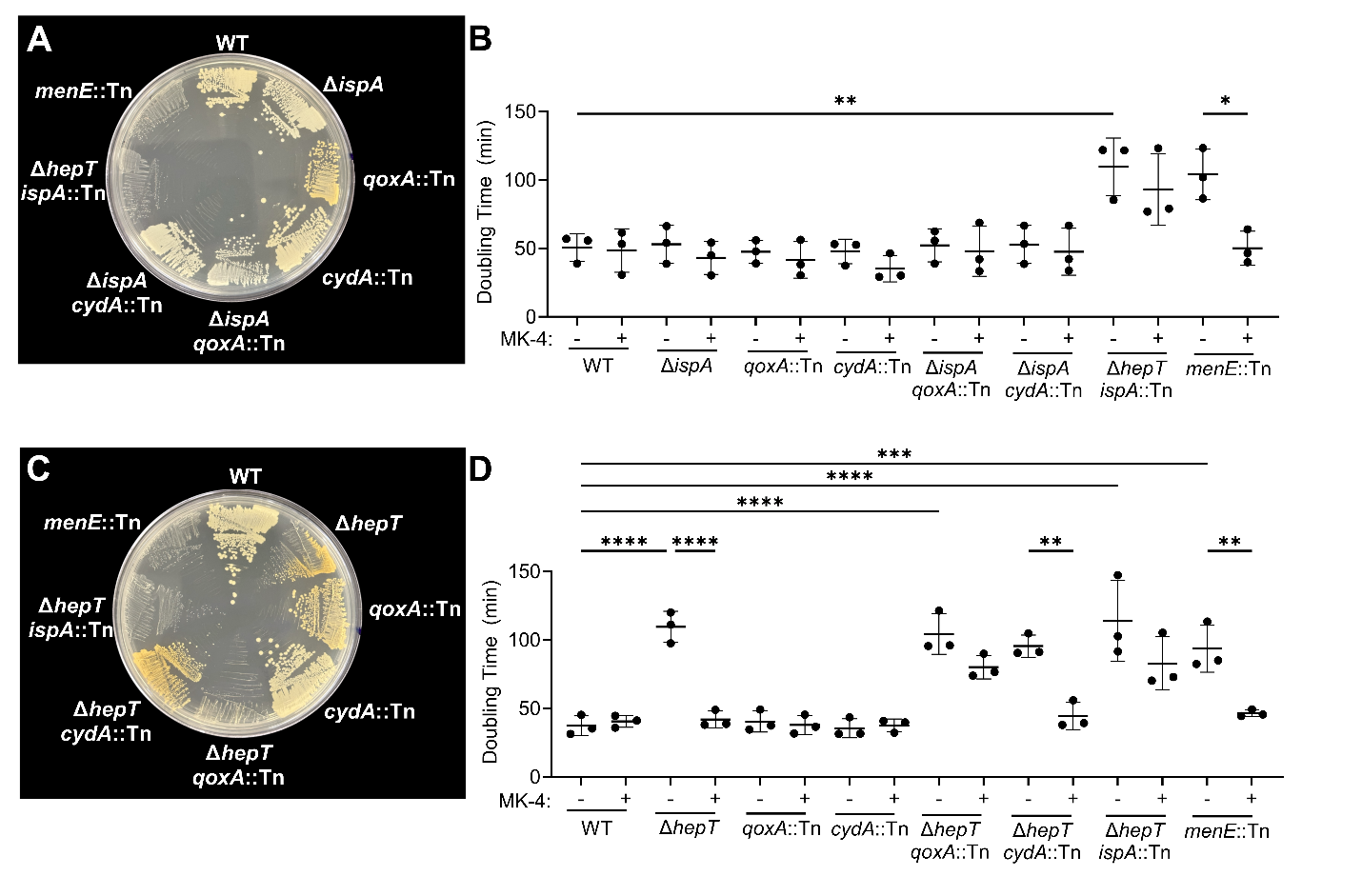


**Figure S5: The Δ*hepT* *qoxA*::Tn double mutant and the Δ*hepT ispA*::Tn double mutant fail to respond to MK-4 supplementation.**

**A** and **C.** The indicated strains streaked onto TSA plates and incubated overnight. **B** and **D.** Doubling times of the indicated strains measured in minutes (min) in TSB or TSB supplemented with 12.5 µM MK-4. Error bars represent one standard deviation from the mean. Data are the average of three independent biological replicates performed in technical triplicate. Statistical significance was determined via one-way ANOVA. *, **, ***, and **** represent p-values of <0.05, <0.01, <0.001, and <0.0001, respectively.


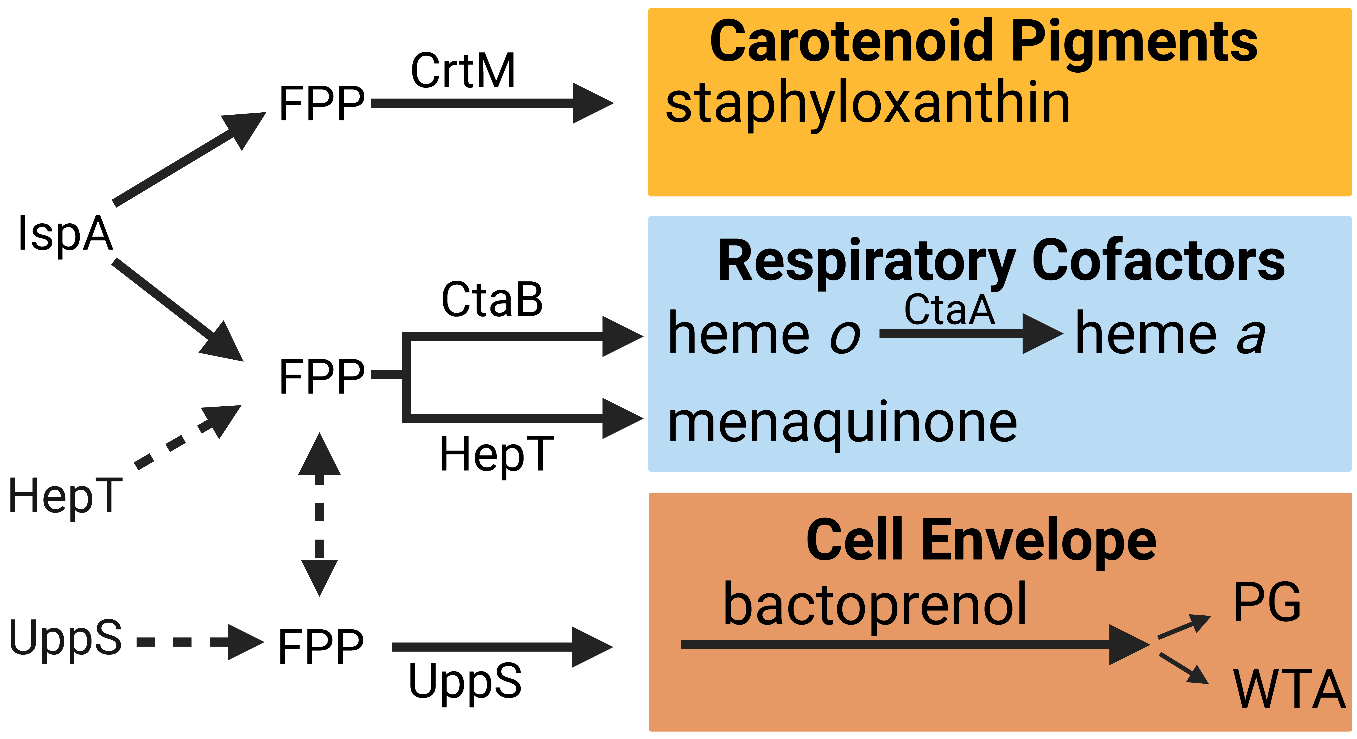


**Figure S6: A revised segregated model of *S. aureus* isoprenoid synthesis based on the characterization of the *ispA hepT* double mutant.**

The updated model of *S. aureus* isoprenoid synthesis presenting multiple foci of FPP production. In this model IspA generates FPP used to make respiratory cofactors and bactoprenol but is the only enzyme capable of producing FPP for staphyloxanthin. Our results support the hypothesis (dashed line) that HepT synthesizes FPP that is preferentially used to generate menaquinone but can also support production of prenylated hemes and possibly bactoprenol. Our results predict (dashed lines) that UppS produces FPP that is used exclusively to synthesize bactoprenol.
